# Does PRP enhance bone integration with grafts, graft substitutes, or implants? A systematic review

**DOI:** 10.1186/1471-2474-14-330

**Published:** 2013-11-21

**Authors:** Alice Roffi, Giuseppe Filardo, Elizaveta Kon, Maurilio Marcacci

**Affiliations:** 1 Nano-Biotechnology Laboratory, Rizzoli Orthopaedic Institute, Via di Barbiano n 1/10, Bologna 40136, Italy; 2 Biomechanics Laboratory, Rizzoli Orthopaedic Institute, Via di Barbiano n 1/10, Bologna 40136, Italy

**Keywords:** PRP, Implant integration, Platelets, Bone, Regenerative medicine

## Abstract

**Background:**

Several bone implants are applied in clinical practice, but none meets the requirements of an ideal implant. Platelet-rich plasma (PRP) is an easy and inexpensive way to obtain growth factors in physiologic proportions that might favour the regenerative process. The aim of this review is to analyse clinical studies in order to investigate the role of PRP in favouring bone integration of graft, graft substitutes, or implants, and to identify the materials for which the additional use of PRP might be associated with superior osseo- and soft tissues integration.

**Methods:**

A search on PubMed database was performed considering the literature from 2000 to 2012, using the following string: ("Bone Substitutes"[Mesh] OR "Bone Transplantation"[Mesh] OR "Bone Regeneration"[Mesh] OR "Osseointegration"[Mesh]) AND ("Blood Platelets"[Mesh] OR "Platelet-Rich Plasma"[Mesh]). After abstracts screening, the full-texts of selected papers were analyzed and the papers found from the reference lists were also considered. The search focused on clinical applications documented in studies in the English language: levels of evidence included in the literature analysis were I, II and III.

**Results:**

Literature analysis showed 83 papers that fulfilled the inclusion criteria: 26 randomized controlled trials (RCT), 14 comparative studies, 29 case series, and 14 case reports. Several implant materials were identified: 24 papers on autologous bone, 6 on freeze-dried bone allograft (FDBA), 16 on bovine porous bone mineral (BPBM), 9 on β-tricalcium phosphate (β-TCP), 4 on hydroxyapatite (HA), 2 on titanium (Ti), 1 on natural coral, 1 on collagen sponge, 1 on medical-grade calcium sulphate hemihydrate (MGCSH), 1 on bioactive glass (BG) and 18 on a combination of biomaterials. Only 4 papers were related to the orthopaedic field, whereas the majority belonged to clinical applications in oral/maxillofacial surgery.

**Conclusions:**

The systematic research showed a growing interest in this approach for bone implant integration, with an increasing number of studies published over time. However, knowledge on this topic is still preliminary, with the presence mainly of low quality studies. Many aspects still have to be understood, such as the biomaterials that can benefit most from PRP and the best protocol for PRP both for production and application.

## Background

Osseointegration is achieved when there is no progressive relative movement between the implant and the bone in direct contact with it [1], and is the result of two complex stages: osteoinduction, the process by which osteogenesis is induced and osteoconduction, the growth of bone on a surface [2]. Osteoinduction is a part of the normal bone healing process and is responsible for the majority of newly formed bone. Osteoconduction also occurs during normal remodelling in bone and depends not only on biological factors, but also on the response to a foreign material [2].

Several bone substitutes materials are currently being applied in clinical practice [3], but none meets all the requirements of an ideal implant. Ideally, to obtain good osseointegration, bone implants should provide four elements: structural integrity; an osteoconductive matrix as a scaffold that permits bone ingrowth; osteogenic cells, which offer the potential to differentiate and facilitate the various stages of bone regeneration; and osteoinductive factors, the mediators that induce the various stages of bone regeneration and repair [3].

Growth factors (GFs) are expressed during different phases of tissue healing and are therefore a key element in promoting tissue regeneration [4]; in fact, GFs carried on orthopaedic devices have been reported to enhance osteoblastic activity and favour implant integration [5,6].

Platelet-rich plasma (PRP) is an inexpensive way to obtain many GFs in physiological proportion and has already been largely applied as a carrier of GFs in different fields of medicine (sports medicine, orthopaedics, dermatology, ophthalmology, plastic and maxillofacial surgery, neurosurgery, urology, and cardiothoracic surgery…) due to its property of favouring tissue healing even in tissues with low healing potential [7-10].

PRP can be defined as a blood derivate where platelets have a higher concentration above baseline levels. In clinical practice PRP has been applied in musculoskeletal treatment, with results reported on cartilage, bone, muscle, tendon and ligament regeneration, and also as an augmentation procedure to favour implant healing, although this aspect has not been largely documented in the literature [7,9]. The first evidence of the clinical benefits of PRP in implant osseointegration was reported in 1998 by Marx et al. [11], who studied 88 patients with mandibular defects treated with platelet concentrate and cancellous cellular marrow bone graft. Results showed that PRP allowed a radiographic graft maturation rate of 1.62 to 2.16 times higher than that without PRP at six months, and also showed greater bone density. Since then the use of PRP has been broadened as an augmentation procedure for several applications.

The aim of this review is to analyse all the existing published clinical studies in order to investigate the role of PRP in favouring integration of bone-graft, bone-graft substitutes, or bone-implants with bone and/or soft tissues, and to identify the materials for which the additional use of PRP might be associated with superior osseo- and soft tissues integration.

## Methods

**Figure 1:**
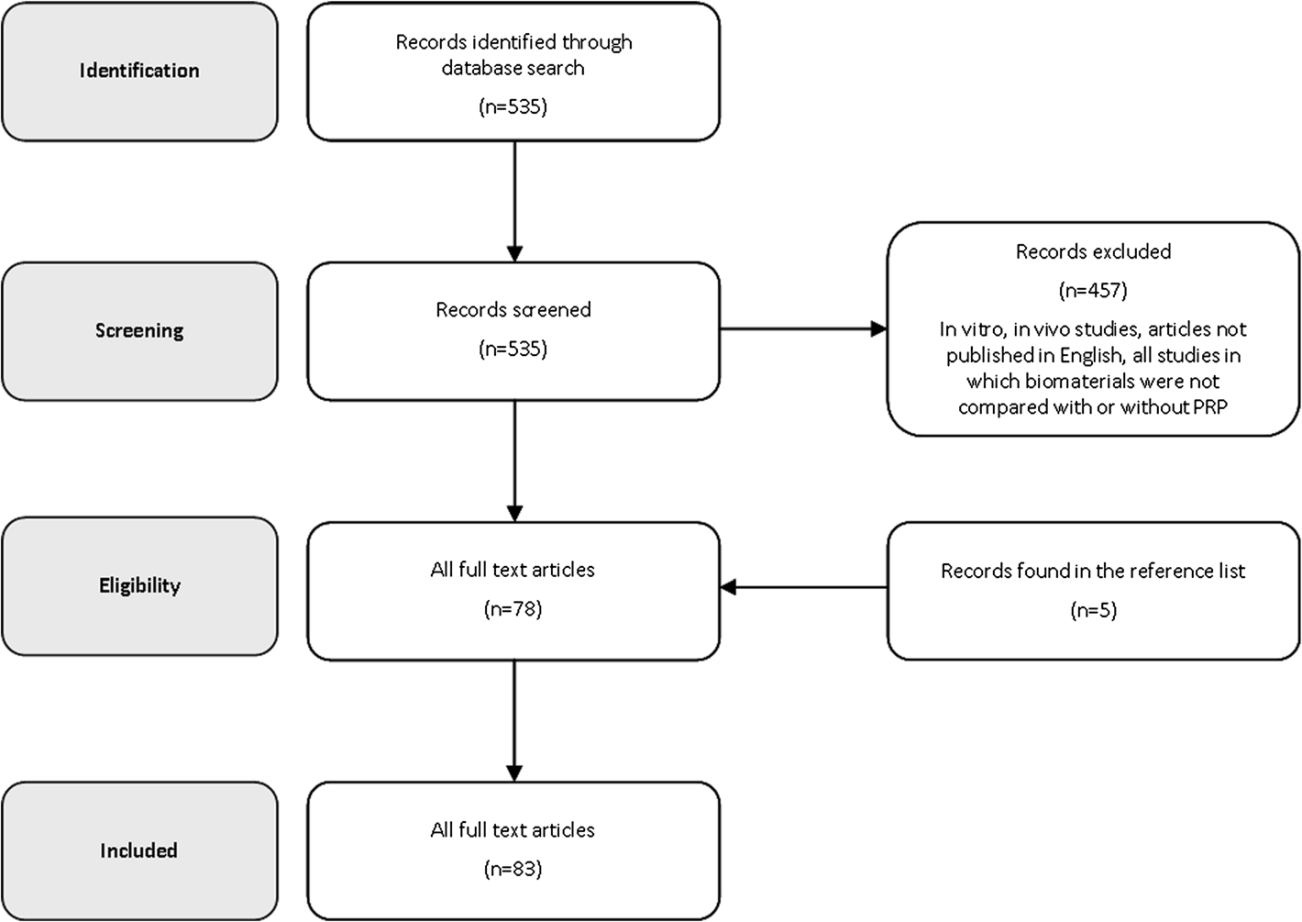
Flow diagram of the systematic review process.

A search was performed on the PubMed database considering the literature from 2000 to 2012, using the following string: ("Bone Substitutes"[Mesh] OR "Bone Transplantation"[Mesh] OR "Bone Regeneration"[Mesh] OR "Osseointegration"[Mesh]) AND ("Blood Platelets"[Mesh] OR "Platelet-Rich Plasma"[Mesh]). After abstracts screening, the full-texts of selected papers were analyzed and the papers found from the reference lists were also considered for the literature analysis of this review (Figure 1).

The search focused on clinical applications documented in studies in the English language: the levels of evidence for the literature analysis were I to III.

Papers were classified according to the level of evidence and biomaterial in order to understand the potential of PRP to favour the osseointegration of different types of biomaterials. In particular, RCTs have been analysed in the text and summarized in Table 1, whereas comparative trials were synthesize in Table 2. Only papers that compared the results of specific treatments with or without PRP were considered. Those using PRP in all the treated groups and where other factors were the only difference were excluded. All papers documenting PRP augmentation for orthopaedic procedures were described separately to understand the evidence available on its potential in this field.

**Table 1:** Published results on PRP clinical application to favor bone implant integration (R: randomized trial; C: comparative study; +: results in favor of PRP; -: no benefit from PRP; +/-: doubtful results on PRP usefullness for bone scaffold integration)

	**Publication**	**N° pts**	**Study type**	**Results**		**Publication**	**N° pts**	**Study type**	**Results**
**Autologous bone**	Khairy NM [12]	15	R	+	**BPBM**	Camargo PM [13]	28	C	+/-
	J Oral Maxillofac Surg - 2012					Int J Per Rest Dent - 2005			
	Wei LC [14]	276	R	+		Hanna R [15]	13	R	+/-
	J Orthop Res - 2012					J Periodontol - 2004			
	Sys J [16]	40	R	–		Lekovic V [17]	52	C	+/-
	Eur Spine J - 2011					J Clin Periodontol - 2003			
	Luaces-Rey R [18]	20	C	–		Camargo PM [19]	18	C	+/-
	Med Oral Patol Oral Cir Bucal - 2010					J Periodontal Res - 2002			
	Badr M [20]	21	R	–	**β-TCP**	Ozdemir B [21]	14	R	–
	Eur J Oral Implantol - 2010					J Biomed Mater Res B - 2012			
	Bettega G [22]	18	C	+		Saini N [23]	20	C	+
	Transfusion - 2009					Indian J Dent Res - 2011			
	Lee C [24]	30	C	+		Harnack L [25]	22	R	–
	Int J Oral Maxillofac Surg - 2009					Clin Oral Investig - 2009			
	Schaaf H [26]	34	R	–		Dori F [27]	28	R	–
	Oral Surg Radiol Endod - 2008					J Periodontol - 2008			
	Consolo U [28]	16	R	+		Yassibag-Berkman Z [29]	25	R	–
	Clin Oral Impl Res - 2006					J Periodontol - 2007			
	Raghoebar GM [30]	30	R	–		Wiltfang J [31]	39	R	+
	Clin Oral Implants Res - 2005					Clin Oral Implants Res - 2003			
**FDBA**	Markou N [32]	24	R	–	**HA**	Menzes LM [33]	60	R	+
	J Periodontol - 2009					Quitessence Int - 2012			
	Piemontese M [34]	60	R	–		Vaishnavi C [35]	20	R	+
	J Periodontol - 2008					J Conserv Dent - 2011			
	Kassolis JD [36]	10	R	+		Okuda K [37]	35	R	+/-
	J Craniofac Surg - 2005					J Periodontol - 2005			
**BPBM**	Anitua E [38]	5	C	+	**BG**	Demir B [39]	29	R	–
	Clin Impl Dent Rel Res - 2012					J Clin Periodontol - 2007			
	Cabbar F [40]	10	C	–	**Ti**	Monov G [41]	10	C	–
	J Oral Maxillofac Surg - 2011					Clin Oral Implants Res - 2005			
	Yilmaz S [42]	20	C	–	**MGCSH**	Kutkut A [43]	16	R	+/-
	J Periodontol - 2011					J Periodontol - 2012			
	Camargo PM [44]	23	C	–	**Biomaterials combination**	Poeschl PW [45]	14	C	+
	J Periodontol - 2009								
						J Oral Maxillofac Surg - 2012			
	Torres J [46]	87	R	+		Inchingolo F [47]	127	C	+
	J Clin Periodontol - 2009					Eur Rev Med Pharmacol Sci - 2012			
	Dori F [48]	24	R	–		Kaushick BT [49]	10	R	+
	J Periodontol - 2007					Indian J Dent Res - 2011			
	Dori F [50]	30	R	–		Torres J [51]	30	R	+
	J Clin Periodontol - 2007					J Clin Periodontol - 2010			

**Table 2:** Comparative studies on PRP clinical application to favor bone-graft, bone-graft substitutes, or bone implant integration

	**Publication**	**Results**		**Publication**	**Results**
**Autologous bone**	Luaces-Rey R [18] Med Oral Patol Oral Cir Bucal - 2010	Bone increase between the third and sixth months, in autologous bone alone or in combination with PRP without any statistical difference	**BPBM**	Camargo PM [13] Int J Per Rest Dent - 2005	Decrease in pocket depth and an increase in clinical attachment level and defect filling, with BPBM, GTR, and PRP in comparison to open flap debridement group
	Bettega G [22] Transfusion - 2009	PRP permitted a 60% reduction in the amount of bone graft required for normal sinus floor augmentation and the bone obtained had the same histological and mechanical properties as the bone obtained by traditional graft		Lekovic V [17] J Clin Periodontol - 2003	Greater pocket reduction, an increase in clinical attachment level, vertical and horizontal defect filling in the BPBM/GTR/PRP group respect to open flap debridement
	Lee C [24] Int J Oral Maxillofac Surg - 2009	PRP acceleration of early bone remodelling, but no significant differences in bone resorption rate between PRP and particulate cancellous bone and marrow (PCBM) vs PCBM alone		Camargo PM [19] J Periodontal Res - 2002	High pocket depth reduction, clinical attachment gain and defect filling in the PRP/BPBM group
**BPBM**	Anitua E [38] Clin Impl Dent Rel Res - 2012	More new vital bone than that of the controls in PRGF-treated samples, and well incorporated bovine into the new bone formation in the PRGF group	**β-TCP**	Saini N [23] Indian J Dent Res - 2011	Clinical and radiographic improvement when PRP was added to β-TCP
	Cabbar F [40] J Oral Maxillofac Surg - 2011	No significant differences in BPBM plus PRP or alone: bone integration and residual graft particles in all patients at histological analysis	**Ti**	Monov G [52] Clin Oral Implants Res - 2005	No statistically significant differences between titanium plus PRP or titanium alone group
	Yilmaz S [42] J Periodontol - 2011	At 12 months, similar results in probing depth reduction, attachment gain, clinical and radiographic bone gain between BPBM plus PPP or plus PRP groups	**Biomaterials combination**	Poeschl PW [53] J Oral Maxillofac Surg - 2012	Significantly better overall graft resorption and increase in bone formation occurred when PRP was added to algae-derived HA, Cgraft and Algipore respect to biomaterials alone
	Camargo PM [44] J Periodontol - 2009	Similar results between BPBM/GTR alone or in combination with PRP in the decrease of probing depth, gain in clinical attachment and bone filling of the defect.		Inchingolo F [54] Eur Rev Med Pharmacol Sci - 2012	At radiographic point of view presence of newly formed bone tissue, well amalgamated with the residual bone in PRP plus autologous bone, anorganic bone material (Bio-Oss, HA) and organic bone substitutes vs autologous bone alone

## Results

The literature analysis showed 83 papers on this topic: 26 randomized controlled trials (RCT), 14 comparative studies, 29 case series, and 14 case reports. The results showed an increasing interest in this topic over time. According to the type of material, several implant types were identified: 24 papers on autologous bone use, 6 on freeze-dried bone allograft (FDBA), 16 on bovine porous bone mineral (BPBM), 9 on β-tricalcium phosphate (β-TCP), 4 on hydroxyapatite (HA), 2 on titanium (Ti), 1 on natural coral, 1 on collagen sponge, 1 on medical-grade calcium sulphate hemihydrate (MGCSH), 1 on bioactive glass (BG), and 18 on a combination of biomaterials. Only 4 papers were related to the orthopaedic field, whereas the majority of the results belonged to clinical applications in oral/maxillofacial surgery.

### Autologous bone

Autologous bone represents the gold standard for bone replacement, because it offers minimal immunological rejection, complete histocompatibility, provides the best osteoconductive and osteoinductive properties, and is inexpensive and easy to obtain [55]. Nevertheless, it also has some drawbacks, such as donor site morbidity, need for general anesthesia or sedation, occasional need for more than one surgical site and limited availability [55]. Several studies have reported its use in combination with PRP to improve bone implant integration.

In 2005 Raghoebar et al. [30] analysed 30 patients that underwent floor augmentation of the maxillary sinus and were randomly assigned to autologous bone graft and PRP or autologous bone alone. No differences between treatments were observed, thus showing no additional value of PRP on implant integration. Conversely, in 2006 Consolo et al. [28] reported the regenerative potential of PRP when used with autologous bone, but this effect appeared to be restricted to shorter treatment times: 16 patients underwent bilateral sinus floor augmentation, using autologous bone on one side and PRP plus autologous bone contralaterally. At 4 months, the PRP group showed higher bone activities documented by histological analysis, but a progressive extinguishment of the PRP effect was recorded after a time of longer than 6–7 months. In 2008 Schaaf et al. [26] showed no significant differences in bone volume and implant failure using autologous bone graft alone or in combination with PRP in 34 sinus floor augmentations. In 2010, Badr et al. [20] used PRP in combination with bone iliac crest graft in maxilla defects: 22 patients were randomly divided into two groups: PRP augmented and controls. No significant differences were detected for implant stability or mean graft resorption and soft tissue healing indices. Only the posterior implant subgroup showed higher stability values, although not clinically significant. Finally in 2012, Khairy et al. [12] evaluated the potential benefit of PRP in conjunction with autologous bone for maxillary sinus augmentation in 15 patients; autogenous bone alone was used as the control group. PRP improved the handling properties of the graft material but did not improve bone density at 3 months. However, PRP-enriched bone grafts were associated with superior bone density at 6 months.

### Freeze-dried bone allograft

FDBA is derived from the removal of water by the frozen tissue with subsequent vacuum-packing and storing at room temperature for up to 5 years [3].

Kassolis et al. [36] in 2005 investigated the use of PRP in combination with FDBA in 10 patients who underwent bilateral maxillary subantral sinus augmentation. The subjects were randomly assigned to FDBA plus PRP or FDBA plus resorbable membrane of polytetrafluoroethylene (e-PTFE). Biopsies were obtained 4.5 to 6 months after treatment and revealed a significantly higher percentage of sinus vital tissue in the PRP group. A lower percentage of residual graft particles and a higher rate of bone formation, although not significant, were detected in the PRP treatment group. In 2008, Piemontese et al. [34] performed a double-blinded RCT on 60 patients with infrabony osseous defects derived from chronic periodontitis and treated with FDBA and PRP or FDBA alone. One year after treatment, both groups showed similar significant changes in the gingival index, bleeding on probing, probing depth, clinical attachment level and radiographic parameters, but a greater probing depth reduction and clinical attachment gain were seen in the PRP group. However, with regards to bone regeneration, PRP did not seem to give any additional value. Similarly, in 2009 Markou et al. [32] compared FDBA plus PRP with FDBA alone in 24 patients with severe chronic periodontitis. At six months the two treatment groups were comparable and the percentage of defect filling did not differ significantly.

### Bovine porous bone mineral

BPBM is a xenograft prepared by protein extraction of bovine bone, which results in a structure similar to human cancellous bone and has the ability to enhance bone formation [56]. The advantages of xenografts include their relative abundant supply, ease of use, and potentially favourable clinical performance. Although rare, one drawback in its use concerns the possible risk of disease transmission, such as bacterial, viral, and prion transmission [57].

The combination of PRP and BPBM has been applied by many research groups, mainly focusing on periodontal regenerative therapy.

In 2004, Hanna et al. [15] reported their experience in the treatment of periodontal intrabony defects using BPBM and PRP: 13 patients were randomly assigned to BPBM or BPBM plus PRP groups. After 6 months significant benefits with both treatments were revealed, but in the PRP group better results were found in probing reduction and clinical attachment level. In 2007, Dori et al. [48] investigated the use of BPBM/GTR alone or in combination with PRP for the treatment of 24 intrabony defects related to chronic periodontal disease, and showed no differences in any of the studied parameters. Similar results were reported in another RCT performed by the same author, who in 2007 analysed 30 patients treated with BPBM/GTR/PRP or BPBM/GTR alone: PRP did not give any additional value [50]. Conversely, a good clinical outcome was reported by Torres et al. [46] two years later: 87 patients underwent sinus floor augmentation with BPBM alone or in combination with PRP. Histological analysis revealed that bone regeneration was significantly higher in sites treated with PRP and BPBM, whereas graft resorption was similar in both groups.

### Ceramics

Ceramics have been widely used for their osteoconductive properties. Most calcium phosphate ceramics currently under investigation are synthetic and composed of HA (Ca_10_[PO_4_]_6_[OH_2_]), TCP (Ca_3_[PO_4_]_2_), or a combination of the two [3]. Clinically good short-term results have been reported for bone grafting with ceramic bone substitute materials [58].

#### β-Tricalcium phosphate

In 2003, Wiltfang et al. [31] analysed 39 patients undergoing sinus floor elevation with β-TCP alone or in combination with PRP. At 6 months, the formation of new bone was 8-10% higher when PRP was added, even if it did not accelerate the degradation of the ceramic bone substitute. Four years later, Yassibag-Berkman et al. [29] tested the efficacy of β-TCP alone or in combination with PRP and GTR in 25 patients: the defects were randomly and equally assigned to three groups, β-TCP alone, β-TCP with PRP and β-TCP with PRP and GTR. No statistically significant differences in clinical and radiographic measurements were observed among the groups.

In 2008, Dori et al. [27] investigated the use of PRP and β-TCP in subjects with intrabony defects caused by chronic periodontal disease: 28 patients were randomly divided into two groups, PRP plus β-TCP and GTR vs β-TCP plus GTR. No significant differences between the groups were observed, thus no additional value was provided by PRP. One year later, Harnack et al. [25] reported the results of an RCT including 22 patients with intrabony defect caused by periodontitis treated with β-TCP in combination with PRP or alone. Both groups showed a similar clinical improvement, thus suggesting that PRP did not enhance or improve bone healing or β-TCP integration. More recently, Ozdemir et al. [21] treated 14 patients with chronic periodontitis (a total of 28 defects) using PRP plus β-TCP or β-TCP alone: no statistically significant differences between the two groups in clinical and radiographic values were observed.

#### Hydroxyapatite

In 2005, Okuda et al. [37] reported promising results using HA together with PRP: 35 patients were treated with HA alone or in combination with PRP and they were evaluated at one year. Significant changes in probing reduction, clinical attachment gain and vertical relative attachment gain suggested that PRP may led to more favourable results compared to HA alone.

More recently in 2011, Vaishnavi et al. [35] showed good results evaluating 20 subjects randomly assigned to four treatments: HA, PRP, HA plus PRP, and no substitutes. Radiographic evaluation showed complete bone regeneration in group I at 1 year, group II at the end of 9 months, group III at the end of 6 months, whereas the last group showed no satisfactory bone regeneration, even at the end of one year. This suggests that PRP favours better and faster bone regeneration combined with HA. Finally, in 2012 Menezes et al. [33] treated 60 intraosseous defects derived from chronic periodontitis with PRP and HA or a mixture of HA and saline. The 1-year results showed no significant changes when compared with baseline; however, the 4-year results indicated that the test group exhibited a more favourable clinical improvement in intraosseous periodontal defects.

#### Bioactive glass

Among various subgroups of alloplastic bone grafts, BG is a kind of bioactive ceramic [59] consisting of SiO2, CaO, Na2O and P2O5. It has been suggested that bioactive glasses bond to bone without a fibrous connective tissue interface [60]. Schepers and Ducheyne [60] evaluated bone growth around bioactive glass particles in dog bone defects in comparison to hydroxylapatite particles, and reported that narrow-size (300– 355 mm) BG has an osteostimulatory effect besides its osteoconductive properties. Moreover, in soft and hard tissue measurements no significant differences were reported between demineralized freeze-dried bone allografts (DFDBA) and BG grafted sites [61]. In 2007, Demir et al. [39] randomly treated 29 intra-bony defects with either PRP/BG or BG alone, and found no additional benefit in the reduction of pocket depth, clinical attachment gain, and defect filling.

### MGCSH

MGCSH is a material that has a long history of clinical use, thanks to its biocompatibility and rapid and complete resorption, although these properties can sometimes be a drawback in the healing process. MGCSH can be used as a carrier to deliver GFs. In 2012, Kutkut et al. [62] reported promising results with PRP and MGCSH: after extraction of a tooth 16 patients received a combination of MGCSH/PRP (test group) or collagen resorbable plug dressing material (control group). The rate of new vital bone after 3 months of healing was 66.5% in the test group compared to 38.3% in the control group. Moreover, PRP enhanced rapid bone healing with respect to PRP-free collagen resorbable graft, but the difference in the material used in the study group prevents a true assessment of the role of PRP.

### Biomaterial combinations

Biomaterials can also be used in combination to incorporate all the favourable material properties in one implant.

In 2010, Torres et al. [63] investigated the role of PRP in alveolar ridge augmentation with Ti-mesh and BPBM. Higher bone augmentation and no Ti-mesh exposure were seen in the PRP group. One year later, Kaushick et al. [41] investigated the use of PRP together with HA/b-TCP: defects of 10 patients were randomly assigned to test (PRP/HA/b-TCP) or saline-HA/b-TCP. PRP permitted a greater reduction in probing pocket depth, gain in attachment level and amount of radio density with respect to the control group.

The present analysis suggests that PRP might not be indicated for b-TCP implants, controversial results are obtained with autologous bone, whereas a better potential seems to lie in the augmentation of HA implants. In particular, only 2 out of 6 papers showed good results for the integration of β-TCP, 5 out of 10 for autologous bone, 1 out of 3 for FDBA, 2 out of 11 for BPBM (4 with less clear evidence of PRP effect), whereas 2 out of 3 papers showed good results for the integration of HA (the third one showed some benefit but with less clear evidence).

### Orthopaedic papers

Only a few orthopaedic papers were found in the present search. In 2007, Smrke et al. [43] described the use of allogenic PRP in combination with autologous cancellous bone for the treatment of a tibial fracture and delayed union after insufficient initial osteosynthesis in a 50-year-old type 2 diabetic man. After 6 months, the graft was incorporated, the bone defect was fully bridged and full weight-bearing capacity was achieved. No side effects and no signs of platelet or HLA I antibodies were reported. In the same year, Dallari et al. [51] also showed good results in 33 patients undergoing high tibial osteotomy to treat genu varum. Subjects treated with lyophilized bone chips and PRP, with or without bone marrow stromal cells showed better osseointegration and faster bone healing. In 2011 Sys et al. [49] assessed both the clinical and radiological effect of PRP with autogenous bone in posterior lumbar interbody fusion. Forty patients were randomly treated with autogenous bone alone or in combination with PRP; the subjects were examined at 3, 6, 12, and 24 months postoperatively. The radiographic outcome showed uneventful osseous healing in all patients with no significant differences, but clinical improvement was more pronounced (even if not significantly) in patients who received autografts with PRP. More recently, Wei et al. [45] investigated the use of the same construct to treat 276 calcaneal fractures: the subjects were randomly divided into 3 groups: autogenous bone alone; allograft bone with PRP; and allograft alone. Results showed that PRP augmented the favourable outcome of allografts in the management of displaced calcaneal fractures: at 12 months no significant differences were found between 3 groups, but at 24 and 72 months the results of autologous bone and the allograft with PRP were similar and both were significantly better than the allograft alone.

## Discussion

This systematic research has shown a growing interest in this biological treatment approach as augmentation procedure to favour integration of bone-graft, bone-graft substitutes, or bone implants with bone and soft tissues, by documenting an increasing number of published studies over time. However, knowledge on this topic is still preliminary, as demonstrated by the presence of low quality studies due to weak methodology, small number of patients and short-term follow-up.

The orthopaedic and oral/maxillofacial implants sector forms a significant portion of the biomedical industry and represents a combined $2.8 billion market [47]. The clinical need in all of these areas is justified by increasing prevalence of physically active lifestyles and higher expectations of quality of life in older age groups as well as the ageing population affected by problems of bone healing. Success in the application of an implant depends on many and interconnected factors, that Wang et al. identified as surgeon, patient, and implant factors [6]. However, implant failure may be prevented by adding many coadjuvant agents, which may enhance implant osseointegration potential and restore bone tissue function. Among these, PRP is emerging as a powerful tool for soft and hard tissue healing, thanks to the GFs contained in platelet alpha-granules.

The search showed that among RCT and comparative papers, 16 reported favourable results for PRP augmentation, 18 obtained no significant difference with or without PRP and 6 underlined the doubtful role of PRP.

The great heterogeneity of clinical outcomes can be also explained by the different PRP products that have been used: several different procedures have been described to obtain PRP, thus implying qualitative and quantitative differences among substances (number of platelets concentrated, leukocyte content,..) [64]. Weibrich et al. [65] showed *in vivo* that PRP seems to be able to activate the osseous regeneration processes under optimized conditions, but these are not completely understood and require further studies. PRP activation is another source of variability: some authors do not activate platelets, whereas others use autologous thrombin, calcium chloride, and even physical methods or biomaterials [7]. Finally, applicative protocols can vary widely in terms of amount of substance, number of administrations and timing.

Another controversial point that adds a new variable to this specific type of application concerns the identification of a material that seems to benefit best from PRP augmentation: the lack of comparison between healing potential of biomaterials and PRP, differences in study design and in defects sizes, and low number of patients studied are the main problems that hamper the drawing of conclusions.

The present analysis of RCT and comparative studies showed that a combination of autogenous bone and PRP led to good results only in 5/10 trials, and only 1 of 3 and 2 of 11 studies reported a good clinical outcome using FDBA and BPBM, respectively. A clinical comparative trial published in 2008 and performed by Czuryszkiewicz-Cyrana [16] described a better clinical outcome when PRP was added to autologous bone compared to the addition to β-TCP, thus confirming the present literature findings: only 2 of 6 b-TCP trials found a positive role of PRP during osseointegration. HA implants seem to be the ideal candidate since good clinical outcomes were achieved in all the papers described and promising results, but less conclusive findings, have been reported with combinations of biomaterials. Finally, only one paper was found for both BG and MGCSH with contrasting results: PRP does not add any value when used in combination with BG, whereas it seems to provide positive and faster results with MGCSH.

**Figure 2:**
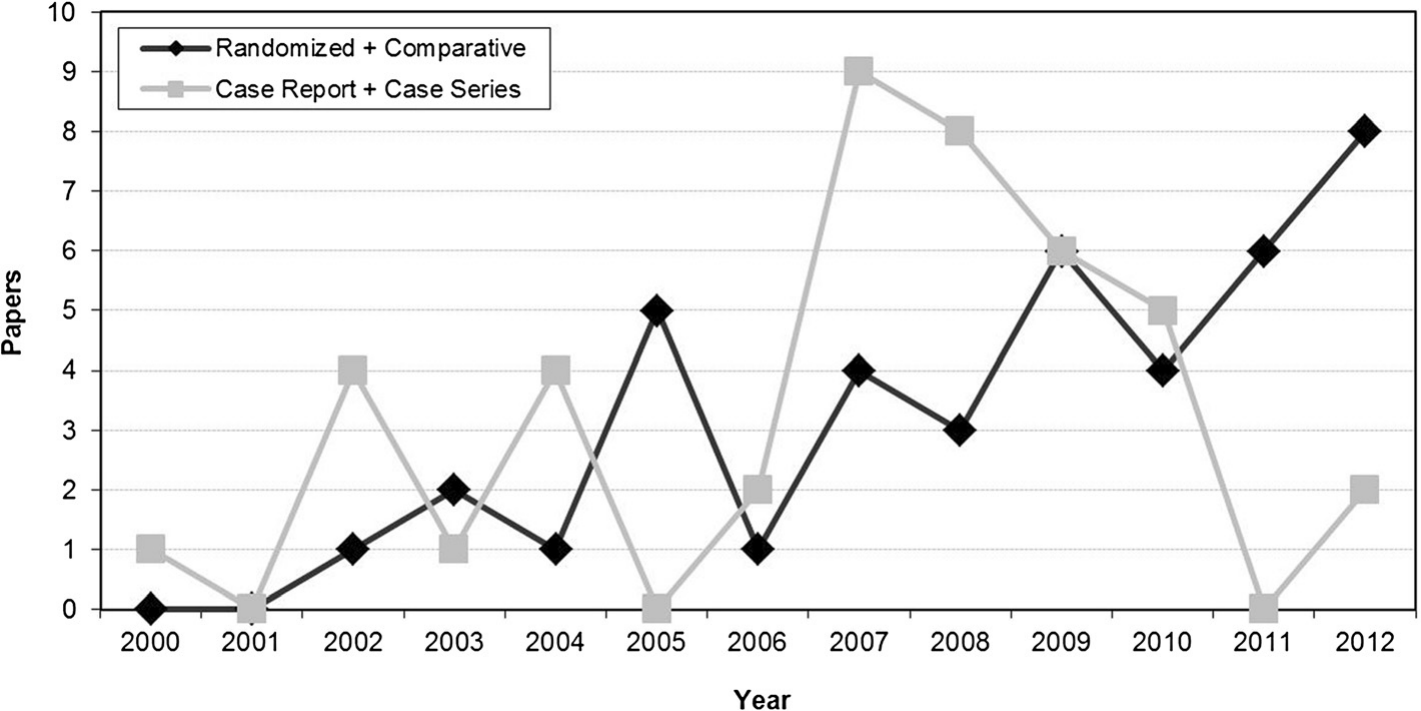
**The literature analysis shows a growing interest on this PRP application.** In particular an increasing number of high-quality trials is highlighted.

Summarizing, for most of the documented applications we do not have enough evidence to draw clear conclusions on the role of PRP as an augmentation procedure. Fortunately, the number of high quality studies are increasing over time, compared to case series and case reports, as shown in Figure 2, and hopefully in the near future some of the many still open questions might be answered. The present analysis suggests that PRP might not be indicated for b-TCP implants, controversial results are obtained with autologous bone, whereas a better potential seems to lie in the augmentation of HA implants. One aspect to be considered is that, besides having a good effect or lack of effect, it does not seem that PRP had a negative effect. However, the weakness of the literature in this field and preclinical findings of a potentially deleterious effect [14] suggest caution and applying PRP as an augmentation procedure only in controlled studies until more evidence will give us better indications on the safety and potential of this biological treatment approach.

An emerging aspect that could represent a further possible application modality of this biological treatment is the potential use of PRP in combination with other bioactive molecules involved in bone metabolism cascade, as bone morphogenetic proteins (BMPs) [66]. It is well known that these molecules can induce bone formation in a variety of indications [67], and recent findings showed that PRP combined with human recombinant BMP2 may promote bone formation in bony defects [67]. This seems to be a promising development of PRP use as augmentation procedure and might provide a fascinating approach to explore in the near future for the enhancement of osteointegration. However, as for all the various applications analysed in this systematic review, the real potential of this blood derivative still needs to be studied and robust trials are required before an indiscriminate application of PRP in the clinical practice.

## Conclusions

Systematic research showed a growing interest in this treatment approach for the integration of bone-graft, bone-graft substitutes, or bone implants, with an increasing number of published studies over time. However, knowledge on this topic is still preliminary, and the few studies available are mainly of low quality.

We do not have enough evidence to draw clear conclusions on the role of PRP as an augmentation procedure: among RCT and comparative papers, 16 reported favourable results, 18 obtained no significant difference with or without PRP and 6 underlined the doubtful role of PRP.

With regards to materials type, PRP might not be indicated for b-TCP implants, controversial results are obtained with autologous bone, whereas a better potential seems to lie in the augmentation of HA implants.

However, several aspects have to be clarified, such as what biomaterials can benefit the most from PRP and what is the best protocol for PRP both for production and application. Randomized controlled trials are needed to support the potential of this treatment approach and the advantages and disadvantages of PRP as an augmentation procedure to favour implant integration.

## Abbreviations

RCT: Randomized controlled trials; FDBA: Freeze-dried bone allograft; BPBM: Bovine porous bone mineral; β-TCP: β-tricalcium phosphate; HA: Hydroxyapatite; Ti: Titanium; MGCSH: Medical-grade calcium sulphate hemihydrate; BG: Bioactive glass; GFs: Growth factors; PRP: Platelet-rich plasma; PCBM: Particulate cancellous bone and marrow; GTR: Guided tissue regeneration; PRGF: Plasma rich in growth factors.

## Competing interests

The authors declare that they have no competing interests.

## Authors’ contributions

Dr. AR: literature analysis, manuscript writing. Dr. GF: literature analysis, manuscript writing. Dr. EK: supervisor and editing. Prof. MM: supervisor, senior consultant, editing. All authors read and approved the final manuscript.

## Pre-publication history

The pre-publication history for this paper can be accessed here:

http://www.biomedcentral.com/1471-2474/14/330/prepub
